# Complete Obstruction of Endotracheal Tube in an Infant with a Retropharyngeal and Anterior Mediastinal Abscess

**DOI:** 10.1155/2017/1848945

**Published:** 2017-02-14

**Authors:** Dennis B. Thapa, Nathaniel H. Greene, Andrea G. Udani

**Affiliations:** Department of Anesthesiology, Duke University School of Medicine, Durham, NC, USA

## Abstract

Intraoperative ventilatory failure is not an uncommon complication; however, acute endotracheal obstruction by a foreign body or blood clot can be difficult to quickly discriminate from other causes. Once the diagnosis is made, quick action is needed to restore ventilation. The ultimate solution is to exchange the endotracheal tube; however, there can be other ways of resolving this in situations where reintubation would be difficult or unsafe. This case report discusses such an event in an infant with multiple airway challenges including a retropharyngeal and anterior mediastinal abscess. We have also formulated a pathway based on various case reports involving complete ETT obstruction.

## 1. Introduction

Although intraoperative ventilatory failure is not an uncommon anesthetic complication, acute endotracheal obstruction by a foreign body or blood clot can be difficult to quickly discriminate from other causes [1]. Such causes to be included in the differential diagnosis include dislodgement or displacement of the endotracheal tube, obstruction from mucous plugs or biting down, pneumothorax, equipment malfunction, or breath stacking. Once the diagnosis of obstruction is made, quick action is needed to restore ventilation. The ultimate solution is to exchange the endotracheal tube; however, there can be other ways of resolving this in situations where reintubation would be difficult or unsafe. This case report discusses such an event in a patient with multiple airway challenges.

## 2. Case Report

**Figure 1:**
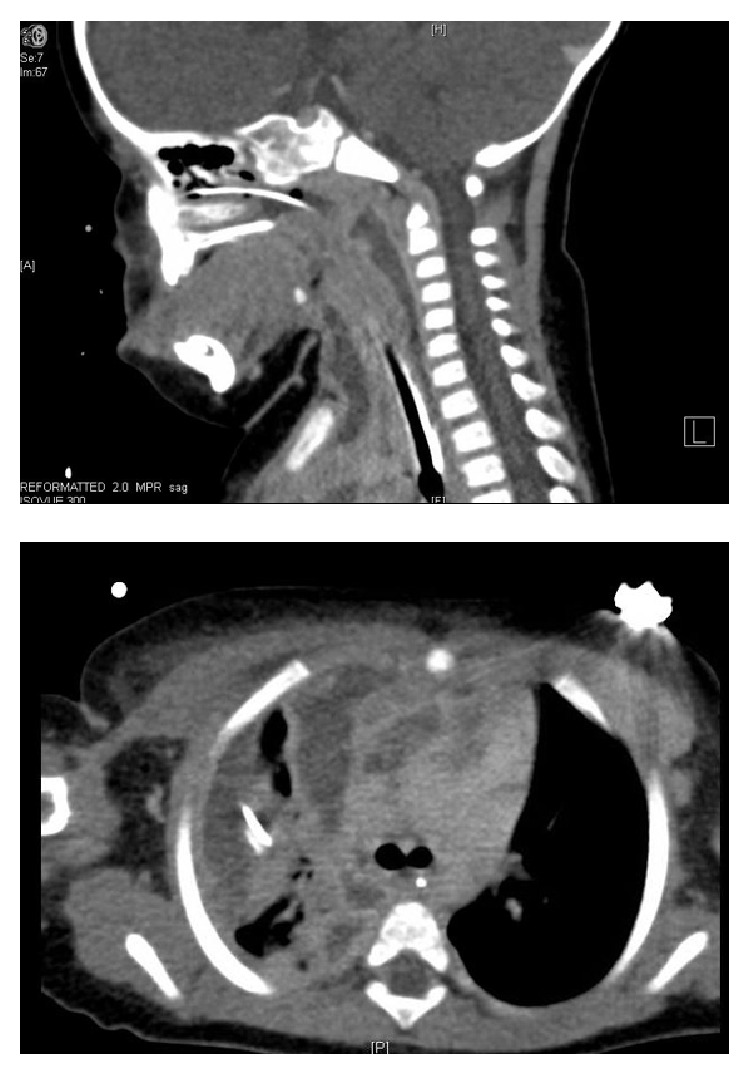
Sagittal and transverse CT images.

A previously healthy 8-month-old female (9 kg) presented with acute onset of fever, noisy breathing, and a swollen right neck. A CT scan (Figure 1) was concerned for a retropharyngeal abscess which extended to the right neck. A large anterior mediastinal abscess was apparent with an associated large empyema in the right chest. The patient was taken for emergent drainage and thoracoscopic decortication.

**Figure 2:**
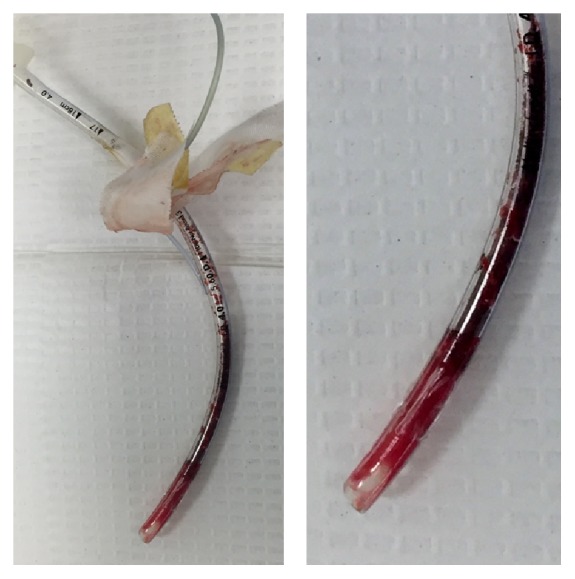
Blood impacted endotracheal tube.

Throughout the case, the patient had brief episodes of hypoxia followed by decreases in heart rate which resolved after manual ventilation. After conclusion of the thoracoscopic procedure, the patient was hypoxic with oxygen saturations down to 40%, had minimal breath sounds, and became increasingly hypercarbic. It was difficult to deliver tidal volumes greater than 15–30 cc with peak inspiratory pressures over 50 mmHg. Attempts to pass a suction catheter through the ETT were unsuccessful. The available fiberoptic scope was unable to fit in the patient's 4.0 ETT, as were any available exchange catheters. An intraoperative chest X-ray showed proper placement of the ETT. There was a high suspicion for a clogged ETT and the decision was to exchange the ETT. A pediatric Glidescope showed severely distorted airway anatomy with blood and purulent contents. As the patient's heart rate dropped (despite resuscitative efforts with epinephrine), direct laryngoscopy was done and the ETT was exchanged with a 4.0 cuffed tube. The patient returned to normal tidal volumes with 100% oxygen saturation. On examination, the old ETT had large blood impaction (Figure 2).

## 3. Conclusion

**Figure 3:**
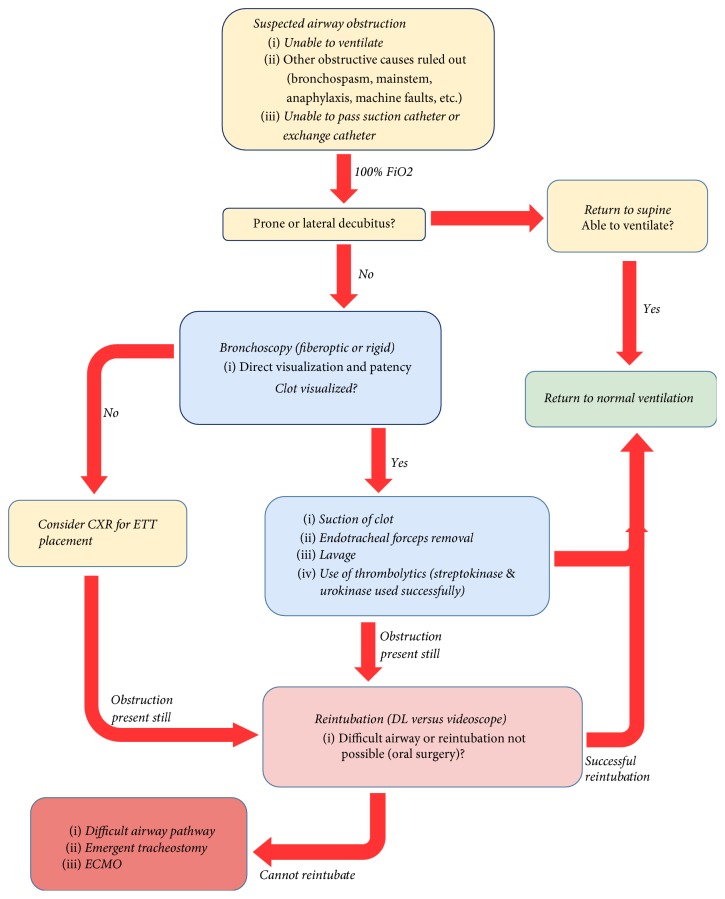
Proposed flowchart for suspected complete ETT obstruction.

Our case illustrates how quickly an obstructed ETT can progress. There have been case reports which have shown various techniques in dealing with blood clot impaction in the ETT [2]. Some methods include aspiration via bronchoscopy, careful use of thrombolytic agents, and repositioning of the patient [3]. Once obstruction is suspected, nearly all reports indicated an inability to pass a suction catheter, making it a diagnostic tool for patency. Two case reports showed relief of obstruction from the prone or lateral position to supine position. Over 95% of all case reports since 1982 used rigid or flexible bronchoscopy. Six cases report the use of streptokinase/urokinase to dissolve the blood impaction [4]. Using the case reports of the last 30+ years, a common algorithm can be devised for treatment and management of a clot obstructed ETT (Figure 3). However, when these options are eliminated, as shown by this case, one must always rely on clinical judgement and experience for guidance.
